# Monoclinic modification of *N*-benzyl­propan-2-aminium chloride

**DOI:** 10.1107/S160053681001456X

**Published:** 2010-04-28

**Authors:** Mehrdad Pourayoubi, Hossein Eshtiagh-Hosseini, Monireh Negari

**Affiliations:** a Department of Chemistry, Ferdowsi University of Mashhad, Mashhad, 91779, Iran

## Abstract

In the title salt, C_10_H_16_N^+^·Cl^−^, the cations and anions are linked by two N—H⋯Cl hydrogen bonds, forming a centrosymmetric tetramer.

## Related literature

For the ortho­rhom­bic modification, see: Pourayoubi & Negari (2010 ▶).
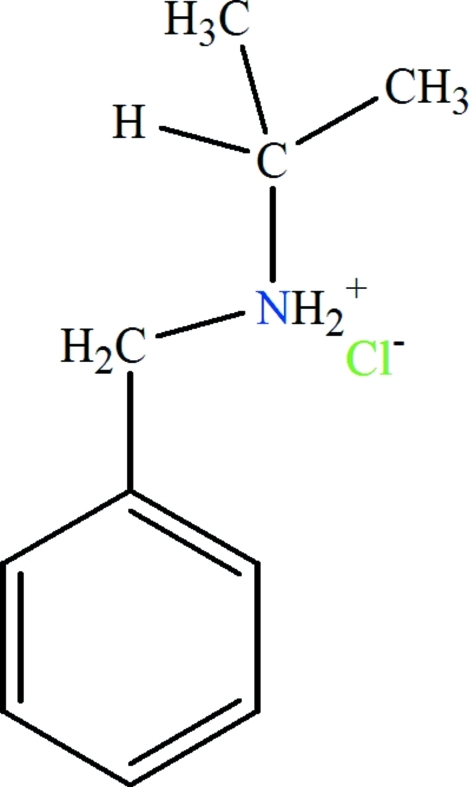


## Experimental

### 

#### Crystal data

C_10_H_16_N^+^·Cl^−^
*M* *_r_* = 185.69
Monoclinic, 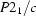

*a* = 9.9566 (7) Å
*b* = 15.5072 (10) Å
*c* = 7.2179 (5) Å
β = 111.112 (1)°
*V* = 1039.63 (12) Å^3^
*Z* = 4
Mo *K*α radiation
μ = 0.32 mm^−1^
*T* = 120 K
0.26 × 0.26 × 0.11 mm

#### Data collection

Bruker SMART 1000 CCD area-detector diffractometer
Absorption correction: multi-scan (*SADABS*; Bruker, 1998 ▶) *T*_min_ = 0.922, *T*_max_ = 0.966
15855 measured reflections
3008 independent reflections
2303 reflections with *I* > 2σ(*I*)
*R*_int_ = 0.032

#### Refinement

*R*[*F* ^2^ > 2σ(*F* ^2^)] = 0.048
*wR*(*F* ^2^) = 0.102
*S* = 1.00
3008 reflections
111 parameters
H-atom parameters constrained
Δρ_max_ = 0.67 e Å^−3^
Δρ_min_ = −0.26 e Å^−3^

### 

Data collection: *SMART* (Bruker, 1998 ▶); cell refinement: *SAINT-Plus* (Bruker, 1998 ▶); data reduction: *SAINT-Plus*; program(s) used to solve structure: *SHELXS97* (Sheldrick, 2008 ▶); program(s) used to refine structure: *SHELXL97* (Sheldrick, 2008 ▶); molecular graphics: *SHELXTL* (Sheldrick, 2008 ▶); software used to prepare material for publication: *SHELXTL*.

## Supplementary Material

Crystal structure: contains datablocks I, global. DOI: 10.1107/S160053681001456X/ng2758sup1.cif

Structure factors: contains datablocks I. DOI: 10.1107/S160053681001456X/ng2758Isup2.hkl

Additional supplementary materials:  crystallographic information; 3D view; checkCIF report

## Figures and Tables

**Table 1:** Hydrogen-bond geometry (Å, °)

*D*—H⋯*A*	*D*—H	H⋯*A*	*D*⋯*A*	*D*—H⋯*A*
N1—H1*NA*⋯Cl1^i^	0.90	2.25	3.1517 (14)	176
N1—H1*NB*⋯Cl1	0.90	2.32	3.2099 (14)	170

Symmetry code: (i) 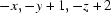.

**Fig. 1.**
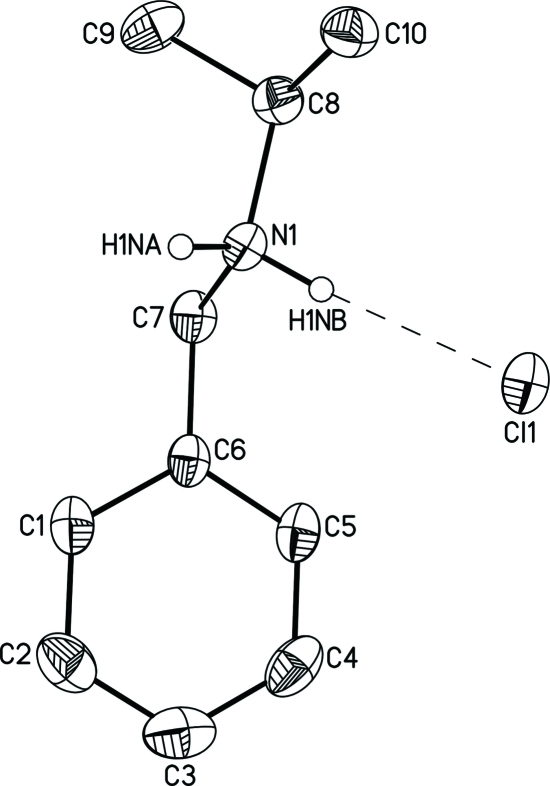
A general view of the title salt, showing the atom-numbering scheme. Displacement ellipsoids are drawn at the 50 % probability level. N(1)—H(1NB)···Cl(1) bond is shown by dash line.

## supplementary crystallographic information

### Comment

In the previous work, the structure determination of orthorhombic polymorph of *N*-benzylpropan-2-aminium chloride (Pourayoubi & Negari, 2010) has been investigated; we report here on the crystal structure of title compound (Fig. 1), a monoclinic polymorph of this salt. The cations and anions are linked together *via* two different N—H···Cl hydrogen bonds to form a centrosymmetric tetramer, in which two Cl^-^ anions act as a bridge between two C_10_H_16_N^+^ cations. The previously reported structure contains an extended zigzag chain arrangement of cations and anions *via* two different N—H···Cl hydrogen bonds.

### Experimental

The title compound is a by-product of the preparation of P(O)[OC_6_H_5_][N(CH_2_C_6_H_5_)(CH(CH_3_)_2_)]Cl [from the reaction between P(O)[OC_6_H_5_]Cl_2_ and NH(CH_2_C_6_H_5_)(CH(CH_3_)_2_), with 1:2 mole ratio] in CCl_4_.

### Refinement

All hydrogen atoms were calculated from geometrical point of view with exception of H1NA and H1NB, which were located from difference Fourier maps. The H atoms were refined in isotropic approximation in riding model with the Uiso(H) parameters equal to 1.2 Ueq(C,*N*), 1.5 Ueq(*C*-methyl), where U(C,*N*) are respectively the equivalent thermal parameters of the carbon and oxygen atoms to which corresponding H atoms are bonded.

### Crystal data

**Table:**

C_10_H_16_N^+^·Cl^−^	*F*(000) = 400
*M**_r_* = 185.69	*D*_x_ = 1.186 Mg m^−^^3^
Monoclinic, *P*2_1_/*c*	Mo *K*α radiation, λ = 0.71073 Å
Hall symbol: -P 2ybc	Cell parameters from 5439 reflections
*a* = 9.9566 (7) Å	θ = 2.2–29.9°
*b* = 15.5072 (10) Å	µ = 0.32 mm^−^^1^
*c* = 7.2179 (5) Å	*T* = 120 K
β = 111.112 (1)°	Prism, colorless
*V* = 1039.63 (12) Å^3^	0.26 × 0.26 × 0.11 mm
*Z* = 4	

### Data collection

**Table:**

Bruker SMART 1000 CCD area-detector diffractometer	3008 independent reflections
Radiation source: fine-focus sealed tube	2303 reflections with *I* > 2σ(*I*)
graphite	*R*_int_ = 0.032
φ and ω scans	θ_max_ = 30.0°, θ_min_ = 2.2°
Absorption correction: multi-scan (*SADABS*; Bruker, 1998)	*h* = −13→14
*T*_min_ = 0.922, *T*_max_ = 0.966	*k* = −21→21
15855 measured reflections	*l* = −10→10

### Refinement

**Table:**

Refinement on *F*^2^	Primary atom site location: structure-invariant direct methods
Least-squares matrix: full	Secondary atom site location: difference Fourier map
*R*[*F*^2^ > 2σ(*F*^2^)] = 0.048	Hydrogen site location: mixed
*wR*(*F*^2^) = 0.102	H-atom parameters constrained
*S* = 1.00	*w* = 1/[σ^2^(*F*_o_^2^) + (0.022*P*)^2^ + 1.240*P*] where *P* = (*F*_o_^2^ + 2*F*_c_^2^)/3
3008 reflections	(Δ/σ)_max_ < 0.001
111 parameters	Δρ_max_ = 0.67 e Å^−^^3^
0 restraints	Δρ_min_ = −0.26 e Å^−^^3^

### Special details

**Table:**

Geometry. All esds (except the esd in the dihedral angle between two l.s. planes) are estimated using the full covariance matrix. The cell esds are taken into account individually in the estimation of esds in distances, angles and torsion angles; correlations between esds in cell parameters are only used when they are defined by crystal symmetry. An approximate (isotropic) treatment of cell esds is used for estimating esds involving l.s. planes.
Refinement. Refinement of *F*^2^ against ALL reflections. The weighted *R*-factor wR and goodness of fit *S* are based on *F*^2^, conventional *R*-factors *R* are based on *F*, with *F* set to zero for negative *F*^2^. The threshold expression of *F*^2^ > σ(*F*^2^) is used only for calculating *R*-factors(gt) etc. and is not relevant to the choice of reflections for refinement. *R*-factors based on *F*^2^ are statistically about twice as large as those based on *F*, and *R*- factors based on ALL data will be even larger.

### Fractional atomic coordinates and isotropic or equivalent isotropic displacement parameters (Å^2^)

**Table:**

	*x*	*y*	*z*	*U*_iso_*/*U*_eq_	
Cl1	0.07253 (4)	0.60195 (3)	0.78749 (6)	0.02540 (11)	
N1	−0.05680 (14)	0.41218 (8)	0.7852 (2)	0.0203 (3)	
H1NA	−0.0578	0.4057	0.9086	0.024*	
H1NB	−0.0137	0.4624	0.7773	0.024*	
C1	0.2525 (2)	0.30399 (11)	1.0254 (3)	0.0290 (4)	
H1A	0.1971	0.2680	1.0768	0.035*	
C2	0.4001 (2)	0.31060 (13)	1.1261 (3)	0.0373 (4)	
H2A	0.4450	0.2790	1.2453	0.045*	
C3	0.4816 (2)	0.36310 (13)	1.0528 (3)	0.0393 (5)	
H3A	0.5825	0.3680	1.1216	0.047*	
C4	0.4152 (2)	0.40859 (12)	0.8782 (3)	0.0363 (4)	
H4A	0.4709	0.4445	0.8270	0.044*	
C5	0.2677 (2)	0.40188 (11)	0.7779 (3)	0.0280 (4)	
H5A	0.2230	0.4334	0.6587	0.034*	
C6	0.18499 (18)	0.34934 (10)	0.8506 (2)	0.0219 (3)	
C7	0.02639 (18)	0.34037 (10)	0.7393 (3)	0.0243 (3)	
H7A	0.0075	0.3398	0.5949	0.029*	
H7B	−0.0069	0.2847	0.7744	0.029*	
C8	−0.21251 (18)	0.41694 (11)	0.6481 (2)	0.0243 (3)	
H8A	−0.2164	0.4219	0.5081	0.029*	
C9	−0.2914 (2)	0.33546 (12)	0.6674 (3)	0.0340 (4)	
H9A	−0.2479	0.2855	0.6276	0.051*	
H9B	−0.3929	0.3399	0.5814	0.051*	
H9C	−0.2841	0.3285	0.8056	0.051*	
C10	−0.27797 (18)	0.49771 (12)	0.7003 (3)	0.0283 (4)	
H10A	−0.2211	0.5481	0.6919	0.042*	
H10B	−0.2779	0.4926	0.8357	0.042*	
H10C	−0.3771	0.5045	0.6070	0.042*	

### Atomic displacement parameters (Å^2^)

**Table:**

	*U*^11^	*U*^22^	*U*^33^	*U*^12^	*U*^13^	*U*^23^
Cl1	0.0337 (2)	0.02088 (18)	0.0252 (2)	−0.00177 (15)	0.01491 (16)	0.00074 (15)
N1	0.0230 (6)	0.0190 (6)	0.0207 (6)	−0.0021 (5)	0.0101 (5)	−0.0017 (5)
C1	0.0358 (9)	0.0237 (8)	0.0296 (9)	0.0017 (7)	0.0144 (7)	0.0058 (7)
C2	0.0380 (10)	0.0320 (10)	0.0354 (10)	0.0086 (8)	0.0053 (8)	0.0012 (8)
C3	0.0274 (9)	0.0318 (10)	0.0553 (13)	0.0026 (7)	0.0109 (9)	−0.0149 (9)
C4	0.0374 (10)	0.0273 (9)	0.0550 (12)	−0.0049 (7)	0.0298 (9)	−0.0060 (8)
C5	0.0385 (9)	0.0208 (8)	0.0309 (9)	0.0011 (7)	0.0201 (8)	0.0013 (7)
C6	0.0293 (8)	0.0153 (7)	0.0245 (8)	0.0024 (6)	0.0139 (6)	−0.0011 (6)
C7	0.0305 (8)	0.0173 (7)	0.0264 (8)	0.0015 (6)	0.0120 (7)	−0.0031 (6)
C8	0.0237 (8)	0.0273 (8)	0.0213 (7)	−0.0013 (6)	0.0075 (6)	−0.0016 (6)
C9	0.0286 (9)	0.0344 (10)	0.0395 (10)	−0.0109 (7)	0.0130 (8)	−0.0092 (8)
C10	0.0236 (8)	0.0308 (9)	0.0308 (9)	0.0034 (6)	0.0102 (7)	0.0013 (7)

### Geometric parameters (Å, °)

**Table:**

N1—C7	1.495 (2)	C5—H5A	0.9500
N1—C8	1.511 (2)	C6—C7	1.498 (2)
N1—H1NA	0.8999	C7—H7A	0.9900
N1—H1NB	0.9001	C7—H7B	0.9900
C1—C6	1.388 (2)	C8—C9	1.520 (2)
C1—C2	1.388 (3)	C8—C10	1.521 (2)
C1—H1A	0.9500	C8—H8A	1.0000
C2—C3	1.382 (3)	C9—H9A	0.9800
C2—H2A	0.9500	C9—H9B	0.9800
C3—C4	1.387 (3)	C9—H9C	0.9800
C3—H3A	0.9500	C10—H10A	0.9800
C4—C5	1.387 (3)	C10—H10B	0.9800
C4—H4A	0.9500	C10—H10C	0.9800
C5—C6	1.389 (2)		
			
C7—N1—C8	114.28 (12)	N1—C7—C6	111.92 (13)
C7—N1—H1NA	110.0	N1—C7—H7A	109.2
C8—N1—H1NA	106.2	C6—C7—H7A	109.2
C7—N1—H1NB	108.4	N1—C7—H7B	109.2
C8—N1—H1NB	108.4	C6—C7—H7B	109.2
H1NA—N1—H1NB	109.5	H7A—C7—H7B	107.9
C6—C1—C2	120.88 (17)	N1—C8—C9	109.96 (14)
C6—C1—H1A	119.6	N1—C8—C10	107.96 (13)
C2—C1—H1A	119.6	C9—C8—C10	112.36 (14)
C3—C2—C1	119.97 (18)	N1—C8—H8A	108.8
C3—C2—H2A	120.0	C9—C8—H8A	108.8
C1—C2—H2A	120.0	C10—C8—H8A	108.8
C2—C3—C4	119.60 (18)	C8—C9—H9A	109.5
C2—C3—H3A	120.2	C8—C9—H9B	109.5
C4—C3—H3A	120.2	H9A—C9—H9B	109.5
C3—C4—C5	120.29 (18)	C8—C9—H9C	109.5
C3—C4—H4A	119.9	H9A—C9—H9C	109.5
C5—C4—H4A	119.9	H9B—C9—H9C	109.5
C4—C5—C6	120.49 (17)	C8—C10—H10A	109.5
C4—C5—H5A	119.8	C8—C10—H10B	109.5
C6—C5—H5A	119.8	H10A—C10—H10B	109.5
C1—C6—C5	118.77 (16)	C8—C10—H10C	109.5
C1—C6—C7	120.82 (15)	H10A—C10—H10C	109.5
C5—C6—C7	120.39 (15)	H10B—C10—H10C	109.5
			
C6—C1—C2—C3	0.2 (3)	C4—C5—C6—C7	−178.39 (15)
C1—C2—C3—C4	−0.3 (3)	C8—N1—C7—C6	168.23 (13)
C2—C3—C4—C5	0.3 (3)	C1—C6—C7—N1	97.96 (18)
C3—C4—C5—C6	−0.3 (3)	C5—C6—C7—N1	−83.50 (18)
C2—C1—C6—C5	−0.2 (3)	C7—N1—C8—C9	62.65 (17)
C2—C1—C6—C7	178.39 (16)	C7—N1—C8—C10	−174.45 (13)
C4—C5—C6—C1	0.2 (2)		

### Hydrogen-bond geometry (Å, °)

**Table:**

*D*—H···*A*	*D*—H	H···*A*	*D*···*A*	*D*—H···*A*
N1—H1NA···Cl1^i^	0.90	2.25	3.1517 (14)	176
N1—H1NB···Cl1	0.90	2.32	3.2099 (14)	170

Symmetry codes: (i) −*x*, −*y*+1, −*z*+2.
